# Full-Length Genome Sequence of a Plaque-Cloned Virulent Porcine Epidemic Diarrhea Virus Isolate (USA/Iowa/18984/2013) from a Midwestern U.S. Swine Herd

**DOI:** 10.1128/genomeA.01049-13

**Published:** 2013-12-19

**Authors:** Hai Hoang, Mary L. Killian, Darin M. Madson, Paulo H. E. Arruda, Dong Sun, Kent J. Schwartz, Kyoungjin J. Yoon

**Affiliations:** Department of Veterinary Microbiology and Preventive Medicine, College of Veterinary Medicine, Iowa State University, Ames, Iowa, USAa; Diagnostic Virology Laboratory, National Veterinary Services Laboratories, U.S. Department of Agriculture, APHIS/VS, Ames, Iowa, USAb; Department of Veterinary Diagnostic and Production Animal Medicine, College of Veterinary Medicine, Iowa State University, Ames, Iowa, USAc

## Abstract

Porcine epidemic diarrhea (PED) was recognized in U.S. swine for the first time in early 2013. A plaque-purified PED virus (PEDV) isolate (USA/Iowa/18984/2013) was obtained from a diarrheic piglet. The isolate is genetically close to other previously reported U.S. PEDVs and recent Chinese PEDVs and was virulent when inoculated into neonatal pigs.

## GENOME ANNOUNCEMENT

Porcine epidemic diarrhea (PED), caused by a member of the genus *Alphacoronavirus* (1–3), was first identified in England in 1971 and later in other countries, such as Belgium, China, Hungary, Italy, Japan, Korea, and Thailand (4–11). In April of 2013, PED emerged in U.S. swine (12) and was detected in swine herds in 18 U.S. states by the end of October (http://www.aasv.org/pedv/PEDV_weekly_report_103013.pdf), causing considerable economic losses. An isolate of PED virus (PEDV) was sought for use in studies to address concerns regarding diagnosis, transmission, pathogenesis, and vaccination.

Virus isolation was attempted from field samples submitted to the Iowa State University Veterinary Diagnostic Laboratory that were positive for PEDV by a real-time reverse transcriptase PCR (RT-PCR) assay (13) using Vero cells (ATCC CCL-81), as previously described (14). A cytopathic virus with coronavirus morphology was isolated from intestinal tissues collected from a 1.5-week-old piglet from a swine farm in Iowa. In the cells that were inoculated with the virus, the virus induced syncytia and eventually cell death. The presence of PEDV in the inoculated cells was confirmed by immunofluorescence microscopy using a PEDV-specific monoclonal antibody, 6C8 (12). After 3 cell culture passages, the primary isolate was subjected to plaque purification. A plaque-cloned virus isolate was further propagated in the cells for 2 more passages, for a total of 6 cell passages (P6).

The plaque-cloned PEDV P6 isolate (USA/Iowa/18984/2013) was able to cause cytopathic effects at 12 to 18 h postinoculation (hpi) and reach a titer of 10^5.5^ PFU/ml within 48 hpi. Caesarean-born colostrum-deprived piglets orally inoculated with the isolate at a rate of 10^3^ PFU/ml developed severe watery diarrhea and dehydration within 24 hpi and eventually died. Microscopically, intensive immunohistochemical staining of almost all enterocytes for PEDV was observed at 18 hpi, leading to severe villous atrophy at a later time.

The entire genome of the isolate USA/Iowa/18984/2013 was sequenced using next-generation sequencing technology on the Ion Torrent platform (Life Technologies, Austin, TX) as per the manufacturer’s instructions, and the data were assembled using DNAStar NGen based on known PEDV sequences. The genomic RNA of the isolate is 28,039 nucleotides long, excluding the 3′ poly(A) tail. The genomic organization of the isolate is similar to what was previously described (2, 15, 16) and includes a 5′ untranslated region (5′ UTR), open reading frame 1a (ORF1a)/ORF1b, S, ORF3, E, M, N, and a 3′ UTR with a slippery sequence (_12610_TTTAAAC_12616_) in ORF1. There is an insertion between nucleotides 20204 and 20205 in ORF1 that causes a reading frameshift, shortening the replicase polyprotein 1ab (6,649 amino acids long). Phylogenetically, the isolate is 99.8 to 99.9% similar to other U.S. PEDVs reported earlier (12, 15, 17), 97.2 to 99.6% similar to recent Chinese PEDVs (18–27), with AH2012 (GenBank accession no. KC210145) being the closest, and 96.9% similar to the prototype PEDV strain CV777 (1).

In conclusion, the PEDV isolate USA/Iowa/18984/2013 is a virulent strain with a genetic profile similar to those of other U.S. PEDVs reported to date. Such a representative purified virulent PEDV isolate can be a valuable reagent for studying the pathogenesis and immunobiology of PEDV and developing diagnostic reagents and kits, as well as effective vaccines.

### Nucleotide sequence accession number.

The complete genome sequence of PEDV strain USA/Iowa/18984/2013 was submitted to GenBank under the accession no. KF804028.
